# The 3D structure of fibrous material is fully restorable from its X-ray diffraction pattern

**DOI:** 10.1107/S2052252521004760

**Published:** 2021-06-12

**Authors:** Hiroyuki Iwamoto

**Affiliations:** a SPring-8, Japan Synchrotron Radiation Research Institute, 1-1-1 Kouto, Sayo-cho, Sayo-gun, Hyogo 679-5198, Japan

**Keywords:** X-ray fiber diffraction, 3D structure determination, cylindrically averaged Patterson functions

## Abstract

X-ray fiber diffraction is potentially a powerful technique to study the structure of fibrous materials, such as DNA and synthetic polymers. However, only rotationally averaged diffraction patterns can be recorded and it is difficult to correctly interpret them without the knowledge of esoteric diffraction theories. Here we demonstrate that, in principle, the non-rotationally averaged 3D structure of a fibrous material can be restored from its fiber diffraction pattern. The method is a simple puzzle-solving process and in ideal cases it does not require any prior knowledge about the structure, such as helical symmetry. We believe that the proposed method has a potential to transform the fiber diffraction to a 3D imaging technique, and will be useful for a wide field of life and materials sciences.

## Abstract 2

Non-rotationally averaged 3D structures of fibers can be restored from their rotationally averaged diffraction patterns.

## 1. Introduction   

Like electron microscopy, X-ray diffraction (XRD) is a powerful tool for atomic resolution structure analysis. However, structure analysis by XRD is more complicated than visual observation by electron microscopy because X-ray detectors cannot record the phase of scattered waves. Reconstruction of high-resolution images requires both phase and amplitude information of scattered waves. Because of this, the structure analysis by XRD is almost synonymous to the retrieval of the once-lost phase information. Once it is carried out, XRD functions as X-ray microscopy with molecular-to-atomic resolution.

Fortunately, phase-retrieval methods have well been established in X-ray crystallography (Hauptman, 1986 ▸; Taylor, 2010 ▸). Even for non-crystalline XRD, algorithms have been developed to determine the structures of proteins dissolved in solutions (Svergun & Stuhrmann, 1991 ▸; Chacón *et al.*, 1998 ▸; Grant, 2018 ▸) and isolated single particles (coherent diffractive imaging or CDI) (Miao & Sayre, 2000 ▸). However, X-ray fiber diffraction remains as the last frontier of non-crystalline XRD with no established phase-retrieval methods.

Classically, X-ray fiber diffraction contributed to the discovery of the double-helical structure of DNA (Watson & Crick, 1953 ▸) and today it is applied to a very wide range of materials, from biomolecules to synthetic polymers. Modern synchrotron-based fiber diffraction is the only means that can monitor sub-millisecond molecular motions within functioning fibers, *e.g.* in the flight muscle of a flying insect (Iwamoto & Yagi, 2013 ▸).

In the absence of good phase-retrieval methods, however, information extractable from fiber diffraction is limited. A further problem is that one can usually record only rotationally averaged diffraction patterns, from which angular information is missing. Attempts have been made to apply the CDI technique to fibrous materials (Latychevskaia & Fink, 2018 ▸) but it is not well suited because the continuous scattering densities required for CDI (Miao & Sayre, 2000 ▸) are not usually obtained.

Here we demonstrate that, in principle, the non-rotationally averaged 3D structure of a fiber can be restorable from a single rotationally averaged diffraction pattern, without prior knowledge about the symmetry of fiber structure. This is based on the Patterson method (Patterson, 1934 ▸), one of the direct phase-retrieval methods developed in a very early stage of XRD science. The method is based on a simple and clear procedure, and in most cases the 3D structure is uniquely determined. Unlike in CDI, no iterative calculations are required.

## 2. Principle   

When both amplitude and phase are available, the inverse Fourier transformation (*F* ^−1^) of the scattered waves generates the image of the sample at a high resolution, and this is actually the principle of lens-based microscopes. If one performs a Fourier transform on the intensity of the diffraction pattern, one obtains a Patterson function instead. This is an auto-correlation function of the sample structure and is a list of all the vectors that connect any pairs of masses within the sample. A Patterson function can be calculated from a diffraction pattern and if the sample structure is known it can also be calculated from it. The Patterson function for a 3D sample is also a 3D function.

In the case of fiber diffraction, the vectors are rotated around the fiber axis to fall on a single plane, so that the Patterson function is reduced to 2D (the angular information around the fiber axis is lost). This is called the cylindrically averaged Patterson function (CAP) (MacGillavry & Bruins, 1948 ▸). CAPs have been utilized in the analyses of fiber diffraction patterns from DNA (Franklin & Gosling, 1953 ▸), muscle (Namba *et al.*, 1980 ▸; Oshima *et al.*, 2011 ▸), *etc*, and they give some insights into structural symmetry, but their true potential has not been exploited.

The CAP’s true potential is, as we propose here, that the non-rotationally averaged 3D structure of the sample can be restored from it. The vectors listed in the CAP are like the piled pieces of a jigsaw puzzle and our mission is to tile (re-connect) them in the 3D space to finish the puzzle. The rules are simple:

(1) the vectors can only be translated, rotated around the fiber (*z*) axis and inverted with respect to the *x*–*y* plane;
(2) no vectors in the CAP must be left unused;
(3) one may not create any vector that is not listed in the CAP;
(4) a single vector may be used many times, if necessary.

We have developed a computer program that actually solves the puzzles by following the rules listed above. Detailed information about the program is described in the supporting information, and the results of application to model structures are described below.

## 3. Examples of implementation using model structures   

**Figure 1:**
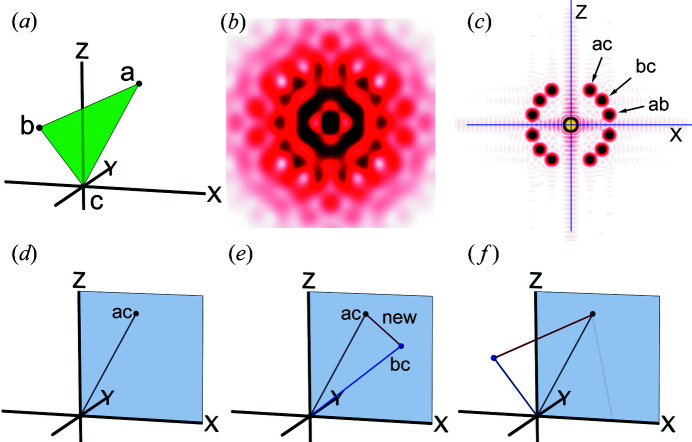
Reconstruction procedure of a 3D structure from its CAP. (*a*) The structure of an object, consisting of only three points (the green plane is for assisting purpose only), freely rotatable around the *z* axis. (*b*) The ‘fiber’ diffraction pattern from the object. (*c*) The CAP of the object, calculated from (*b*). (*d*)–(*f*) The reconstruction procedure. (*d*) One of the vectors (here, vector **ac**) is placed as the starting vector on the *x*–*z* plane (pale blue). (*e*) A second vector (**bc**) is tentatively placed on the *x*–*z* plane and connected to the first vector, and this creates a new incorrect vector (red). (*f*) The second vector is rotated around the *z* axis, and now the new vector coincides with the unused vector **ab** and the reconstruction is finished.

The simplest example is shown in Fig. 1 ▸. Here the sample consists of only three atoms (a, b and c) positioned at the vertices of an equilateral triangle [Fig. 1 ▸(*a*)]. The triangle is freely rotatable around the *z* axis so that its diffraction pattern is rotationally averaged [Fig. 1 ▸(*b*)]. From this diffraction pattern, one obtains the CAP [Fig. 1 ▸(*c*)]. A CAP is symmetrical with respect to the *x* and *z* axes, so the information in just one quadrant is sufficient. There are three vectors in each quadrant, corresponding to vectors **ab**, **ac** and **bc** in the triangle. Now we start to reconstruct the triangle [Fig. 1 ▸(*e*)]. First, we choose one of the vectors (here, **ac**) as a starting vector, and place it on the *x*–*z* plane. Next, we try to connect vector **bc** to vector **ac**. Vector **bc** may start from either end of vector **ac**, and here we tentatively connect vector **bc** to the origin and place it on the *x*–*z* plane. Note here that placing a new vertex in the 3D space creates new vectors [labeled new in Fig. 1 ▸(*d*)], connecting it to all the vertices that are already present. This vector is not listed in the CAP, so rule (3) (see above) is violated. If, however, vector **bc** is rotated around the *z* axis, at an angle, the new vector coincides with vector **ab**. Now all the vectors in the CAP are used and the non-rotationally averaged 3D structure of the triangle is successfully restored [Fig. 1 ▸(*f*), see also Movies S1 and S2 of the supporting information].

**Figure 2:**
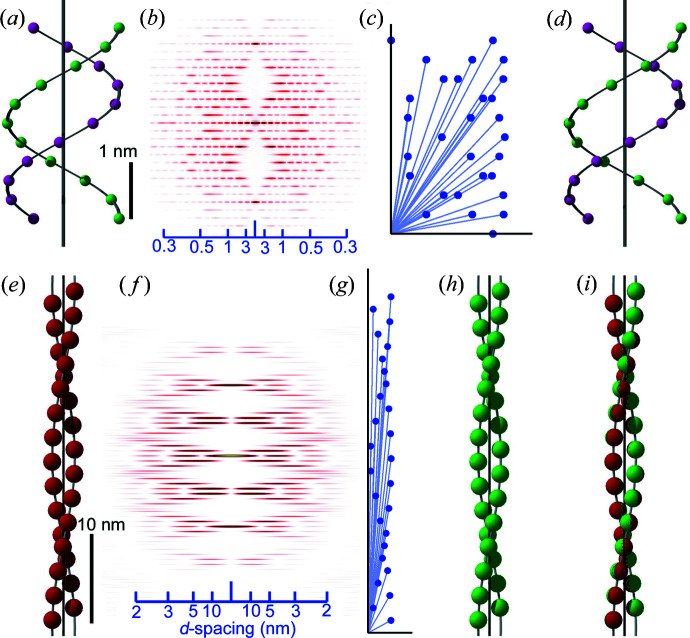
Application of the reconstruction method to helical structures. (*a*), (*e*) The starting model structures of double-stranded DNA (phosphate positions only) and the 28/13 helix of F-actin, respectively. The gray continuous helices and the central axes are shown for assisting purposes. (*b*), (*f*) Expected fiber diffraction patterns, consisting of helix-derived layer-line reflections. (*c*), (*g*) CAPs of the model structures. (*d*), (*h*) The 3D structures reconstructed from the CAPs alone. ( *i* ) A goodness of fit of the original and reconstructed F-actin structures, which depends on the magnitude of permissible errors set in the computer program. The scale bar in (*a*) also applies to (*c*) and (*d*), and the bar in (*e*) also applies to (*g*), (*h*) and (*i*). The values on the scale bars in (*b*) and (*c*) are *d* spacings (nm). Figs. S2 and S3 of the supporting information show how internal vectors in the 3D structures [(*a*) and (*e*)] are picked up in their CAPS [(*c*) and (*g*)].

This is a very simple puzzle game but it works for more complex structures. Fig. 2 ▸ shows examples of the double helix of DNA and the helix of actin filament (28 monomers in 13 turns, or 28/13 helix). For these structures, the CAPs are much more complex, but the computer program quickly finds right answers, as long as an ideal CAP is available (*i.e.* all the vectors are listed and their values are accurate).

The handedness of the helix cannot be determined by diffraction data alone because the diffraction patterns from opposite-handed helices are identical. For this reason, the program may generate helices of opposite handedness or chirality.

## 4. Application to an actual diffraction pattern   

**Figure 3:**
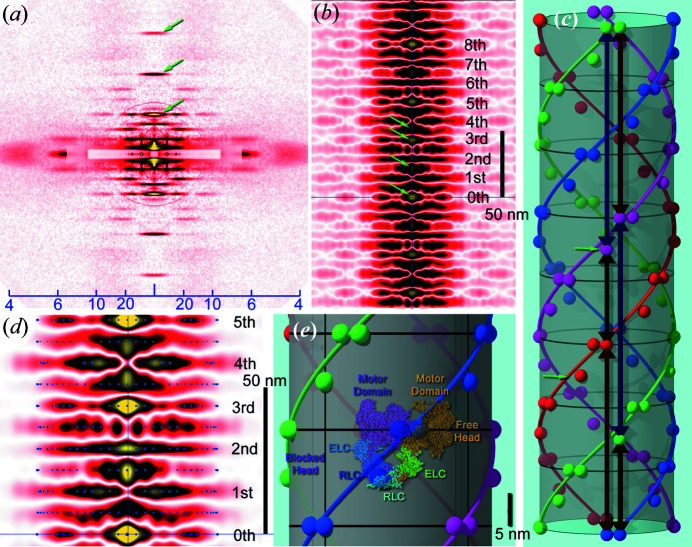
Reconstruction of the 3D structure of myosin filament from an actually recorded diffraction pattern. (*a*) A diffraction pattern from actin-extracted bumblebee flight muscle fibers. The arrows indicate meridional reflections from myosin-head crowns. (*b*) The CAP calculated from the diffraction pattern in (*a*). The numbers indicate the levels of the 14.5 nm crowns of myosin heads. The arrows indicate the peaks on the *z* axis at the 1.5th, 3rd and 3.5th levels of myosin crowns. (*c*) The solved structure of the myosin filament. The black lines on the cylinder indicate the levels of crowns and the small arrows indicate the additional mass lying midway between two neighboring crowns. The green, red and blue double-headed arrows indicate the vertical vectors of 1.5, 3 and 3.5 × 14.5 nm lengths, respectively. (*d*) Superposition of the actually observed CAP and the idealized CAP calculated from the solved structure (blue dots). (*e*) Superposition of the interacting-heads motif (Sweeney, 2018 ▸) and the three-mass unit on the solved structure. The size of the motif was adjusted by using the scale bar in the work of Alamo (2016 ▸) (right). The values on the scale bar in (*a*) are *d* spacings (nm).

In actual recordings, the CAPs calculated from diffraction patterns are not usually ideal for many reasons: fused peaks, missing peaks, false peaks, inaccurate peak positions, *etc*. Even in such difficult cases, some prior knowledge about the symmetry of the structure can help restore the 3D structure from the CAP. Fig. 3 ▸ shows an example: the myosin filament of insect (bumblebee) flight muscle.

Fig. 3 ▸(*a*) shows the small-angle diffraction pattern from demembranated actin-extracted bumblebee (*Bombus*) flight muscle fibers, containing only myosin filaments (see the supporting information for the methods of preparation and recording). Like DNA and actin strands, myosin filaments have a helical structure. As a result, their diffraction pattern also consists of many layer-line reflections, as in Figs. 2 ▸(*b*) and 2 ▸(*f*). A few meridional reflections are also observed and they originate from the 14.5 nm axial repeat of myosin-head crowns.

The CAP calculated from the diffraction pattern [Fig. 3 ▸(*b*)] has many fused peaks. It consists of a stack of densities that occur every 14.5 nm, but densities also occur midway between the two 14.5 nm layers, indicating the presence of mass in the middle of the crowns. In this CAP, the profile at the 8th level of myosin crown is very similar to that at the 0th and the entire pattern is symmetrical with respect to the 4th level. This feature is consistent with the 4-start 8/1.25 helical symmetry of myosin heads in the flight muscle of giant waterbugs (*Lethocerus*) (Tregear *et al.*, 2004 ▸). Therefore, the two insects seem to share the same helical symmetry. Further observation of the CAP shows that there are peaks on the *z* axis at 1.5th, 3rd and 3.5th levels, indicating the presence of vertical vectors with lengths 1.5, 3 and 3.5 × 14.5 nm, respectively. From these features alone, the filament structure is solved without the need for knowing all the vectors, as is shown in Fig. 3 ▸(*c*). At each crown, two masses lie horizontally, separated by 11.25°. In the midway between the crowns, there is an additional mass, slightly offset from the helical path of the masses on the crown.

From this solved structure, an idealized CAP can be calculated, and it is overlaid on the observed CAP at a higher magnification in Fig. 3 ▸(*d*). Its peak positions (blue dots) generally show a good match with the observed densities. Manual fitting of the two CAPs indicates that the radius of the myosin heads from the center is ∼15.3 nm.

Overall, the arrangement of the three masses in the structure in Fig. 3 ▸(*c*) is reminiscent of the interacting-heads motif, in which one of the two heads of a myosin molecule blocks the other head (Zhao *et al.*, 2009 ▸). This motif is commonly observed in many animals (Alamo *et al.*, 2018 ▸). In Fig. 3 ▸(*e*), the atomic model of the motif (Sweeney, 2018 ▸) is superposed on the solved structure. If this superposition is correct, the two masses on each crown correspond to two motor domains and the mass between the crowns corresponds to the light-chain domains. In *Lethocerus* flight muscle, all the domains are at the crown level (Hu *et al.*, 2016 ▸) and this may reflect an inter-species difference.

## 5. Future prospects and conclusions   

This work demonstrates that the non-rotationally averaged 3D structure of a fibrous material is restorable from its diffraction pattern by using a CAP. Under ideal conditions, no prior knowledge is required. If reflections extend to an atomic range (a *d* spacing of 0.3 nm or better), the 3D structure with an atomic resolution will be obtained.

This work presents the principle of the method and deals with the situation in which a CAP correctly lists the internal vectors of the sample. However, CAPs calculated from actual diffraction patterns deviate from it for various reasons besides those listed in Section 4, *e.g.* the curvature of the Ewald sphere and the distortion resulting from projection to flat detectors. In small-angle recordings these effects may be ignored, but in wide-angle recordings these effects are substantial and the data should be corrected before the present method is applied.

The interference between neighboring helices is another issue. Ideally the helices in the sample should be infinitely diluted (as in protein-solution scattering experiments), but for fibrous material it is difficult to do so. In some materials the helices are organized into a lattice, and in this case, inter-helical vectors appear in the CAP in addition to intra-helical vectors. However, inter-helical vectors appear outside the intra-helical vectors [in Fig. 3 ▸(*b*), the weaker densities on both sides are considered to be inter-helical]. One can simply exclude inter-helical vectors from calculations to obtain the structure of a single helix.

One may think that cryoelectron microscopy (cryo-EM) is better suited for high-resolution 3D structure determination than XRD. In principle, however, one should be able to determine atomic resolution 3D structure by using this protocol if one can record diffraction data up to atomic resolution *q* ranges. As briefly stated in the *Introduction* [Sec sec1], XRD is good at dynamic measurements and does not usually requires pre-treatments that may affect the native structure, while in cryo-EM the sample must be cryofixed and is only capable of static measurements. In cryo-EM, a huge number of micrographs must be collected and class averaged, but in XRD, especially that using synchrotron radiation, a single-shot picture often provides sufficient data quality. Thus, high-throughput data acquisition is possible in XRD. Therefore, the present method will provide a quick means for 3D structure assessment on site.

The basic method shown here may be further developed by utilizing unused features of CAPs, *e.g.* the spread of peaks may be used to volume render the restored structure. The imperfectness of real data may be addressed by machine-learning-based inference. It is expected that the publication of this new method will stimulate interested scientists (especially data and computer scientists) to join and accelerate further development of the method. In the end, X-ray fiber diffraction will be an easier-to-use tool to visualize individual atoms or molecules in synthetic and naturally occurring fibers.

## 6. Related literature   

The following references are cited in the supporting information for this article: Iwamoto (2009 ▸), (2017 ▸); Iwamoto *et al.* (2003 ▸); Kawai & Ishiwata (2006 ▸).

## Supplementary Material

Supporting information. DOI: 10.1107/S2052252521004760/ti5020sup1.pdf

Movie S1. A 3D object consisting of 3 points, located at the vertices of an equilateral triangle. From its cylindrically averaged Patterson function (CAP), its 3D structure is restored as demonstrated in Movie S2. DOI: 10.1107/S2052252521004760/ti5020sup2.mp4

Movie S2. Procedure of restoration of non-rotationally averaged 3D structure of the object in Movie S1 from its CAP. DOI: 10.1107/S2052252521004760/ti5020sup3.mp4

Movie S3. Original structure of a double-stranded DNA strand, 11 base pairs (left, phosphate positions only) and the structure restored from its CAP by using the same procedure as in Movie S2 (right). DOI: 10.1107/S2052252521004760/ti5020sup4.mp4

Movie S4. Original structure of a 28/13 helix of actin, 28 monomers (left) and the structure restored from its CAP by using the same procedure as in Movie S2 (right). DOI: 10.1107/S2052252521004760/ti5020sup5.mp4

Movie S5. Structure of the myosin filament of bumblebee flight muscle, solved from the CAP calculated from the diffraction pattern from actin-extracted muscle fibers. Four helical strands of myosin heads are colored differently. DOI: 10.1107/S2052252521004760/ti5020sup6.mp4
