# Supplementary material for: The 3D structure of fibrous material is fully restorable from its X-ray diffraction pattern
Source: IUCrJ. 2021 Jun 12;8(Pt 4):544–8. doi: 10.1107/S2052252521004760 (PMC8256704; doi:10.1107/S2052252521004760)
Supplement: Supplementary file 1 [file m-08-00544-sup1.pdf]

IUCrJ

Volume 8 (2021)

Supporting information for article:

The 3D structure of fibrous material is fully restorable from its X-ray diffraction pattern

Hiroyuki Iwamoto

S1. Further description of structure reconstruction procedure

S1.1. Basic idea

A CAP can be calculated from the structure of a sample consisting of a finite number of vertices distributed in a 3-D space. When a sample consists of n vertices, the number of independent vectors n_v in the CAP (those in the 1st quadrant) is n(n – 1)/2. This does not include zero-length vectors. Either in the Cartesian or cylindrical coordinates, each vertex carries 3 variables to be determined, so that the total number of variables to be determined is 3(n – 1). On the other hand, each vector in the CAP is 2-dimensional, so that it provides 2 equations. Therefore, the total number of the simultaneous equations is n(n – 1). When n $\geqq$ 3, n(n – 1) is always equal to or greater than 3(n – 1), so that the simultaneous equations should be solvable and the phase information should be restorable.

If all of the independent vectors are known, the number of vertices n is calculated by using the following equation:

$$n = (1+ \sqrt{(1 + 8n_v)}) / 2$$

The program determines the x, y and z coordinates of all the vertices in the sample, by using a set of n_v vectors. When the coordinates of all the expected n vertices have been determined, the calculations can be terminated.

When a CAP is calculated from the diffraction pattern, the exact number of vectors is usually unknown, and it is assumed that there are a number of overlapping vectors (i.e., the x and z coordinates on the CAP are identical but connecting different sets of vertices). In this case, attempts to find new vertices are continued even after all the vectors are used.

When no more vertices are found and all the vectors have been used, the reconstructed 3-D structure is regarded correct (a 3-D structure consistent with the CAP has been generated).

S1.2. Specific steps

The specific steps performed in the program are described below. Its code and sample data are available on GitHub (https://github.com/HiIwamoto/FromCAPto3D). The steps are visualized in the form of a flow chart in Fig. S1.

(1) Read a CAP file, which is simply a list of the (x,z) coordinates of the tips of vectors. All the vectors should be in the 1st quadrant. If a vector is identical to one of the already registered vectors, it is not registered.

(2) Pick up one of the registered vectors, and place it on the x-z plane of the 3-D space as the first vector. One end of the vector is placed on the origin.

(3) The other end of this vector is defined as the second vertex. The two vertices are regarded correct, and the coordinates of the rest of the vertices are determined relative to them.

After this, the rest of the vertices are determined by repeating steps (4)-(11).

(4) Choose two vertices that already exist, and to each of them, connect a registered vector.

(5) Check if the z-coordinates of the free ends of the two connected vectors coincide.

(6) If the answer for (5) is yes, further check if the two free ends can be connected. This can be checked if the circular paths along which the free ends can move cross each other.

(7) If the answer for (6) is yes (There are usually two ways of connection. Both will be tested for their validity in the following steps), then the two vectors are connected, and the connection point is tentatively assigned as a new vertex.

(8) When a new vertex is tentatively assigned, it creates vectors with all other existing vertices. It should be verified that all of them are those listed in the CAP. If it has been verified, the new vertex is regarded correct and the process proceed to step (9). If the new vertex creates a vector that is not in a list, the new vertex is regarded incorrect, and the process goes back to (4) and tries another combination of vectors.

(9) The new vertex if formally regarded as a correct vertex.

(10) Check if all the registered vectors has been used. If not, the process goes back to (4) to find more vertices, at least until no unused vector is left. If it is impossible to eliminate unused vectors, it is likely that an incorrect structure has been reconstructed. In that case, calculations are restarted from step (2) under different conditions, e.g., by using an unused vector as the starting vector.

(11) Even if all the registered vectors have been used, it is checked if there are any undiscovered vertices that can be placed without creating unlisted vectors. This is done by going back to the step (4).

If no more new vertices are found, the calculation is finished, and the reconstructed 3-D structure is regarded correct (consistent with the CAP).

S2. Materials and methods of diffraction recordings from muscle fibers

S2.1. X-ray diffraction recording

Bumblebees (*Bombus ignitus*) were collected at or near the campus of SPring-8, and their thoraces were half-split and the flight muscle fibers in them were demembranated in a 50% mixture of a relaxing solution and glycerol, as described (Iwamoto, 2017). The fibers were stored in a 25%:75% mixture of the relaxing solution and glycerol in a -80°C freezer until use. The actin filaments within the flight muscle fibers were removed by treating the fibers with gelsolin (Kawai & Ishiwata, 2006). Bovine gelsolin (Sigma-Aldrich) was dissolved in 2 mM $CaCl_2$ at a 2 mg/ml concentration, and the buffer was exchanged to an extraction buffer (80 mM K-propionate, 10 mM EGTA, 10.4 mM $CaCl_2$, 5 mM $MgCl_2$, 12 mM ATP, 100 mM butanedione monoxime, 20 mM imidazole, pH = 7.2) by using a NAP-5 column (GE healthcare life sciences). 5-drop fractions were collected, and the most concentrated fraction was used for extraction. The extraction was done overnight in a refrigerator, and the solution was then exchanged to a normal relaxing solution before the diffraction experiments. The extent of removal of actin filament was checked by observing the intensities of the actin-based layer line reflections in the X-ray diffraction patterns, as well as by gel electrophoretic patterns. Static X-ray diffraction patterns were recorded in the relaxing solution, in the BL45XU or BL05XU beamlines of SPring-8, as described (Iwamoto, 2009).

S2.2. Processing of X-ray diffraction data

The recorded diffraction patterns were summed and the 4 quadrants were folded to improve the signal-to-noise ratio, and the background scattering was subtracted, as described (Iwamoto et al., 2003). Then the vertical widths of the reflections of interest were reduced to 1 pixel by calculating their integrated intensities along the equator. Other

reflections were deleted. This fine-lined diffraction pattern was then subjected to Fourier transformation to obtain the cylindrically averaged Patterson function (CAP). The intensities on the equator were not used, so that the obtained CAP corresponds to the difference cylindrically averaged Patterson function (Namba et al., 1980).

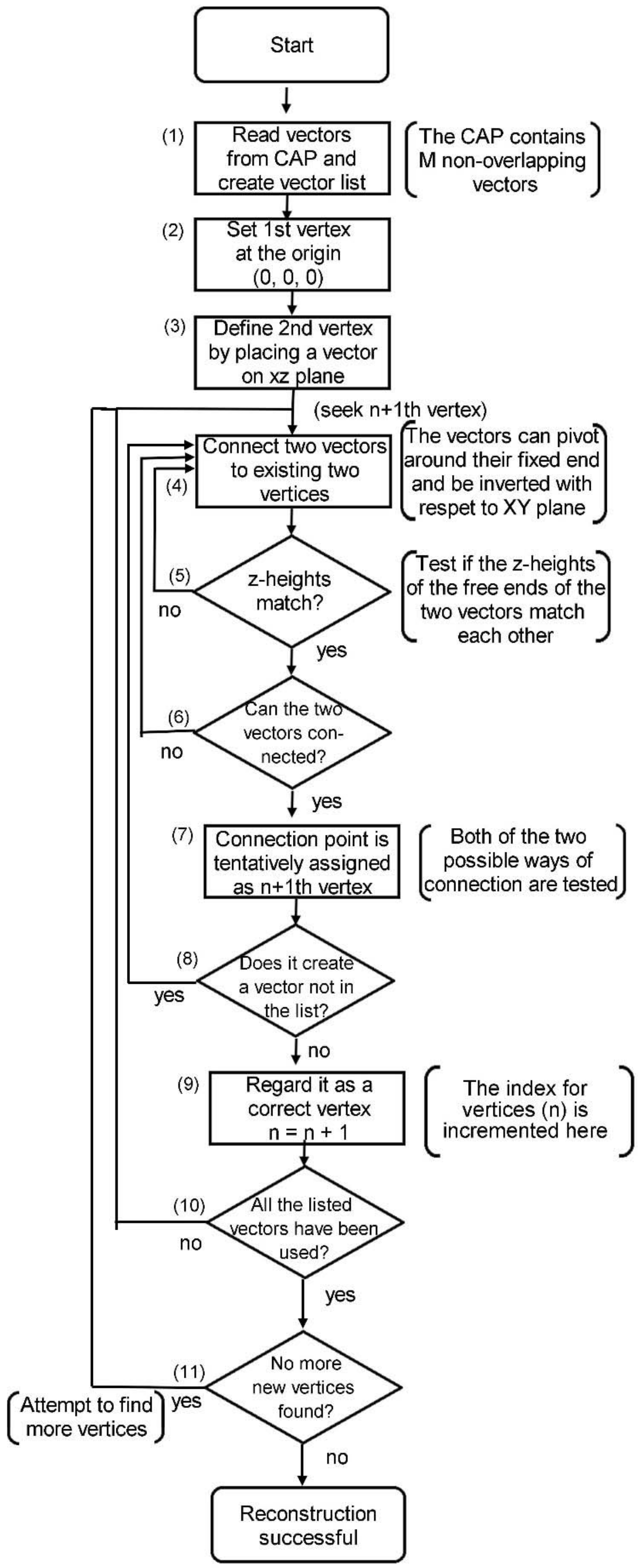
Start
(1)
Read vectors from CAP and create vector list
The CAP contains M non-overlapping vectors
(2)
Set 1st vertex at the origin (0, 0, 0)
(3)
Define 2nd vertex by placing a vector on xz plane
(seek n+1th vertex)
(4)
Connect two vectors to existing two vertices
The vectors can pivot around their fixed end and be inverted with respet to XY plane
(5)
z-heights match?
no
yes
Test if the z-heights of the free ends of the two vectors match each other
(6)
Can the two vectors con-nected?
no
yes
(7)
Connection point is tentatively assigned as n+1th vertex
Both of the two possible ways of connection are tested
(8)
Does it create a vector not in the list?
yes
no
(9)
Regard it as a correct vertex n = n + 1
The index for vertices (n) is incremented here
(10)
All the listed vectors have been used?
no
yes
(11)
No more new vertices found?
yes
Attempt to find more vertices
no
Reconstruction successful

Figure S1 Flow-chart of the reconstruction procedure of 3-D structure from CAP. For details see text.

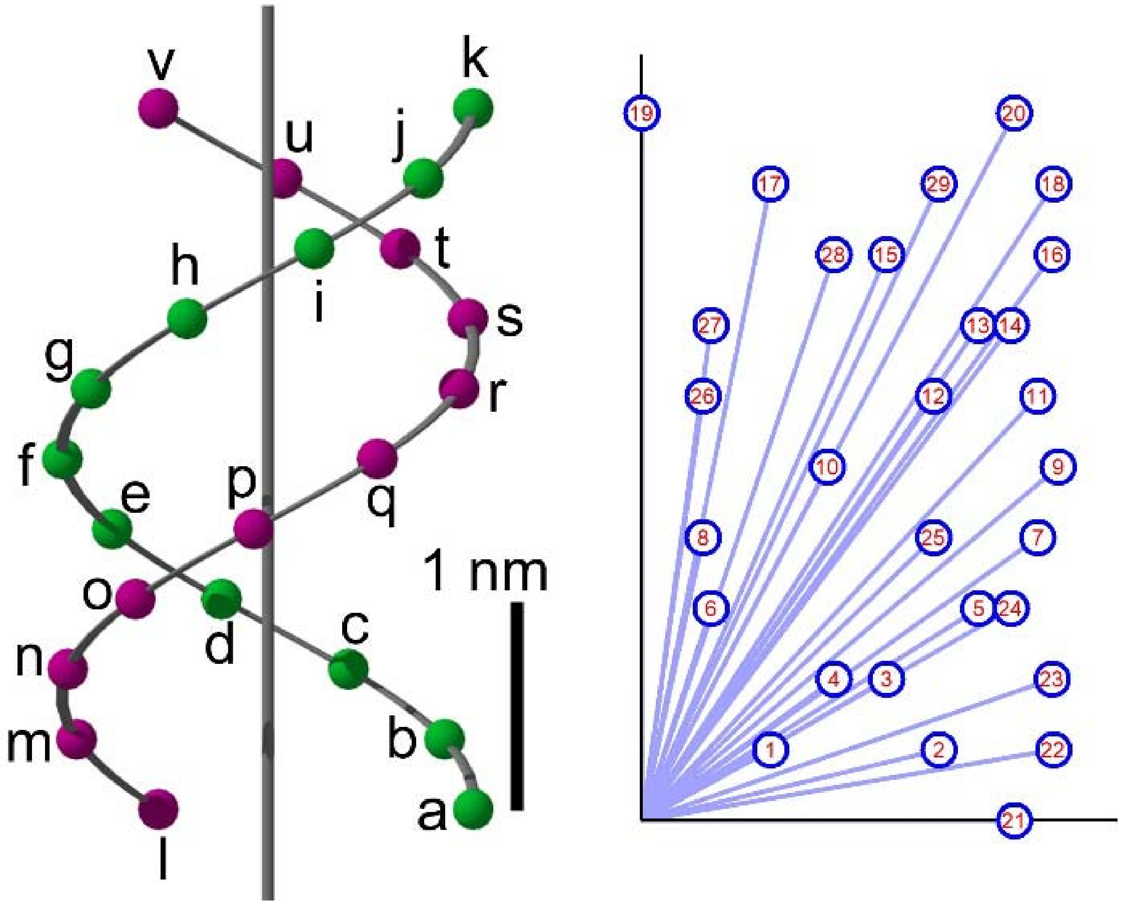

Figure S2 The relationship between the internal vectors in DNA structure (left) and the vectors on the CAP (right). The vectors on the CAP are numbered in red, and the internal vectors represented by each CAP vector are listed below. There are many overlapping vectors, and single number (red) is assigned to them.

CAP vector number (red)	internal vectors
1	a-b,b-c,c-d,d-e,e-f,f-g,g-h,h-i,i-j,j-k,l-m,m-n,n-o,o-p,p-q,q-r,r-s,s-t,t-u,u-v

2	l-b,m-c,n-d,o-e,p-f,q-g,r-h,s-i,t-j,u-k
3	a-c,b-d,c-e,d-f,e-g,f-h,g-i,h-j,i-k,l-n,m-o,n-p,o-q,p-r,q-s,r-t,s-u,t-v
4	l-c,m-d,n-e,o-f,p-g,q-h,r-i,s-j,t-k
5	a-d,b-e,c-f,d-g,e-h,f-i,g-j,h-k,l-o,m-p,n-q,o-r,p-s,q-t,r-u,s-v
6	l-d,m-e,n-f,o-g,p-h,q-i,r-j,s-k
7	a-e,b-f,c-g,d-h,e-i,f-j,g-k,l-p,m-q,n-r,o-s,p-t,q-u,r-v
8	l-e,m-f,n-g,o-h,p-i,q-j,r-k
9	a-f,b-g,c-h,d-i,e-j,f-k,l-q,m-r,n-s,o-t,p-u,q-v
10	l-f,m-g,n-h,o-i,p-j,q-k,a-q,b-r,c-s,d-t,e-u,f-v
11	a-g,b-h,c-i,d-j,e-k,l-r,m-s,n-t,o-u,p-v
12	l-g,m-h,n-i,o-j,p-k
13	a-h,b-i,c-j,d-k,l-s,m-t,n-u,o-v
14	l-h,m-i,n-j,o-k
15	a-i,b-j,c-k,l-t,m-u,n-v
16	l-i,m-j,n-k
17	a-j,b-k,l-u,m-v
18	l-j,m-k
19	a-k,l-v
20	l-k,a-v
21	a-l,b-m,c-n,d-o,e-p,f-q,g-r,h-s,i-t,j-u,k-v
22	a-m,b-n,c-o,d-p,e-q,f-r,g-s,h-t,i-u,j-v
23	a-n,b-o,c-p,d-q,e-r,f-s,g-t,h-u,i-v
24	a-o,b-p,c-q,d-r,e-s,f-t,g-u,h-v
25	a-p,b-q,c-r,d-s,e-t,f-u,g-v

26	a-r,b-s,c-t,d-u,e-v
27	a-s,b-t,c-u,d-v
28	a-t,b-u,c-v
29	a-u,b-v

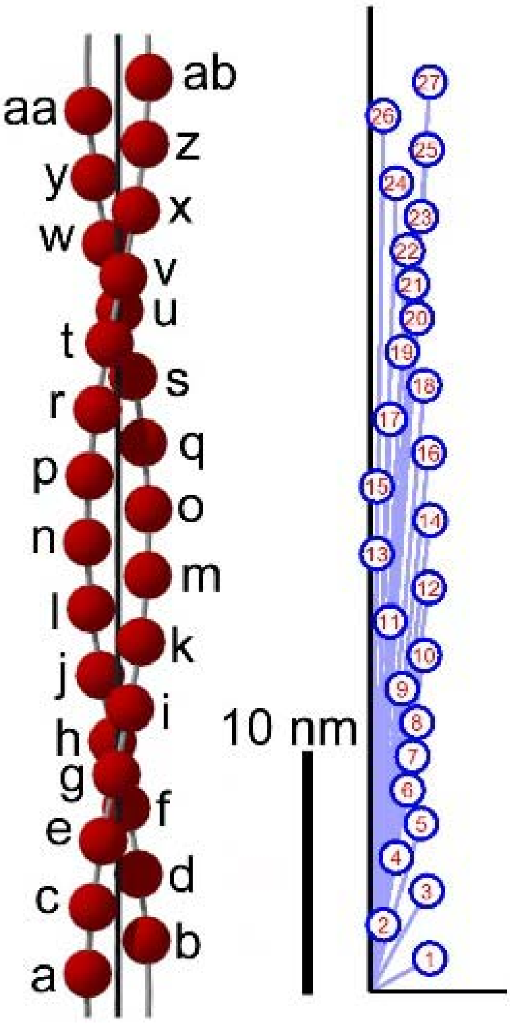

Figure S3 The relationship between the internal vectors in F-actin structure (left) and the vectors on the CAP (right). See legends to Fig. S1 for the way of representation.

CAP vector number (red)	internal vectors
1	a-b,b-c,c-d,d-e,e-f,f-g,g-h,h-i,i-j,j-k,k-l,l-m,m-n,n-o,o-p,p-q,q-r,r-s,s-t,t-u,u-v,v-w,w-x,x-y,y-z,z-aa,aa-ab
2	a-c,b-d,c-e,d-f,e-g,f-h,g-i,h-j,i-k,j-l,k-m,l-n,m-o,n-p,o-q,p-r,q-s,r-t,s-u,t-v,u-w,v-x,w-y,x-z,y-aa,z-ab
3	a-d,b-e,c-f,d-g,e-h,f-i,g-j,h-k,i-l,j-m,k-n,l-o,m-p,n-q,o-r,p-s,q-t,r-u,s-v,t-w,u-x,v-y,w-z,x-aa,y-ab
4	a-e,b-f,c-g,d-h,e-i,f-j,g-k,h-l,i-m,j-n,k-o,l-p,m-q,n-r,o-s,p-t,q-u,r-v,s-w,t-x,u-y,v-z,w-aa,x-ab
5	a-f,b-g,c-h,d-i,e-j,f-k,g-l,h-m,i-n,j-o,k-p,l-q,m-r,n-s,o-t,p-u,q-v,r-w,s-x,t-y,u-z,v-aa,w-ab
6	a-g,b-h,c-i,d-j,e-k,f-l,g-m,h-n,i-o,j-p,k-q,l-r,m-s,n-t,o-u,p-v,q-w,r-x,s-y,t-z,u-aa,v-ab
7	a-h,b-i,c-j,d-k,e-l,f-m,g-n,h-o,i-p,j-q,k-r,l-s,m-t,n-u,o-v,p-w,q-x,r-y,s-z,t-aa,u-ab
8	a-i,b-j,c-k,d-l,e-m,f-n,g-o,h-p,i-q,j-r,k-s,l-t,m-u,n-v,o-w,p-x,q-y,r-z,s-aa,t-ab
9	a-j,b-k,c-l,d-m,e-n,f-o,g-p,h-q,i-r,j-s,k-t,l-u,m-v,n-w,o-x,p-y,q-z,r-aa,s-ab
10	a-k,b-l,c-m,d-n,e-o,f-p,g-q,h-r,i-s,j-t,k-u,l-v,m-w,n-x,o-y,p-z,q-aa,r-ab

11	a-l,b-m,c-n,d-o,e-p,f-q,g-r,h-s,i-t,j-u,k-v,l-w,m-x,n-y,o-z,p-aa,q-ab
12	a-m,b-n,c-o,d-p,e-q,f-r,g-s,h-t,i-u,j-v,k-w,l-x,m-y,n-z,o-aa,p-ab
13	a-n,b-o,c-p,d-q,e-r,f-s,g-t,h-u,i-v,j-w,k-x,l-y,m-z,n-aa,o-ab
14	a-o,b-p,c-q,d-r,e-s,f-t,g-u,h-v,i-w,j-x,k-y,l-z,m-aa,n-ab
15	a-p,b-q,c-r,d-s,e-t,f-u,g-v,h-w,i-x,j-y,k-z,l-aa,m-ab
16	a-q,b-r,c-s,d-t,e-u,f-v,g-w,h-x,i-y,j-z,k-aa,l-ab
17	a-r,b-s,c-t,d-u,e-v,f-w,g-x,h-y,i-z,j-aa,k-ab
18	a-s,b-t,c-u,d-v,e-w,f-x,g-y,h-z,i-aa,j-ab
19	a-t,b-u,c-v,d-w,e-x,f-y,g-z,h-aa,i-ab
20	a-u,b-v,c-w,d-x,e-y,f-z,g-aa,h-ab
21	a-v,b-w,c-x,d-y,e-z,f-aa,g-ab
22	a-w,b-x,c-y,d-z,e-aa,f-ab
23	a-x,b-y,c-z,d-aa,e-ab
24	a-y,b-z,c-aa,d-ab
25	a-z,b-aa,c-ab
26	a-aa,b-ab
27	a-ab

Movie S1

A 3-D object consisting of 3 points, located at the vertices of an equilateral triangle. From its cylindrically averaged Patterson function (CAP), its 3-D structure is restored as demonstrated in Movie S2.

Movie S2

Procedure of restoration of non-rotationally averaged 3-D structure of the object in Movie S1 from its CAP.

Movie S3

Original structure of a double-stranded DNA strand, 11 base pairs (left, phosphate positions only) and the structure restored from its CAP by using the same procedure as in Movie S2 (right).

Movie S4.

Original structure of a 28/13 helix of actin, 28 monomers (left) and the structure restored from its CAP by using the same procedure as in Movie S2 (right).

Movie S5.

Structure of the myosin filament of bumblebee flight muscle, solved from the CAP calculated from the diffraction pattern from actin-extracted muscle fibers. Four helical strands of myosin heads are colored differently.
